# Genistein: A Review on its Anti-Inflammatory Properties

**DOI:** 10.3389/fphar.2022.820969

**Published:** 2022-01-24

**Authors:** Yu Xian Goh, Juriyati Jalil, Kok Wai Lam, Khairana Husain, Chandini Menon Premakumar

**Affiliations:** ^1^ Centre for Drug and Herbal Development, Faculty of Pharmacy, Universiti Kebangsaan Malaysia, Kuala Lumpur, Malaysia; ^2^ Centre for Quality Management of Medicines, Faculty of Pharmacy, Universiti Kebangsaan Malaysia, Kuala Lumpur, Malaysia

**Keywords:** genistein, anti-inflammatory, nuclear factor kappa B, prostaglandin, reactive oxygen species, nitric oxide production, pro-inflammatory cytokines

## Abstract

Nowadays, non-resolving inflammation is becoming a major trigger in various diseases as it plays a significant role in the pathogenesis of atherosclerosis, asthma, cancer, obesity, inflammatory bowel disease, chronic obstructive pulmonary disease, neurodegenerative disease, multiple sclerosis, and rheumatoid arthritis. However, prolonged use of anti-inflammatory drugs is usually accompanied with undesirable effects and hence more patients tend to seek for natural compounds as alternative medicine. Considering the fact above, there is an urgency to discover and develop potential novel, safe and efficacious natural compounds as drug candidates for future anti-inflammatory therapy. Genistein belongs to the flavonoid family, in the subgroup of isoflavones. It is a phytoestrogen that is mainly derived from legumes. It is a naturally occurring chemical constituent with a similar chemical structure to mammalian estrogens. It is claimed to exert many beneficial effects on health, such as protection against osteoporosis, reduction in the risk of cardiovascular disease, alleviation of postmenopausal symptoms and anticancer properties. In the past, numerous *in vitro* and *in vivo* studies have been conducted to investigate the anti-inflammatory potential of genistein. Henceforth, this review aims to summarize the anti-inflammatory properties of genistein linking with the signaling pathways and mediators that are involved in the inflammatory response as well as its toxicity profile. The current outcomes are analysed to highlight the prospect as a lead compound for drug discovery. Data was collected using PubMed, ScienceDirect, SpringerLink and Scopus databases. Results showed that genistein possessed strong anti-inflammatory activities through inhibition of various signaling pathways such as nuclear factor kappa-B (NF-*κ*B), prostaglandins (PGs), inducible nitric oxide synthase (iNOS), proinflammatory cytokines and reactive oxygen species (ROS). A comprehensive assessment of the mechanism of action in anti-inflammatory effects of genistein is included. However, evidence for the pharmacological effects is still lacking. Further studies using various animal models to assess pharmacological effects such as toxicity, pharmacokinetics, pharmacodynamics, and bioavailability studies are required before clinical studies can be conducted. This review will highlight the potential use of genistein as a lead compound for future drug development as an anti-inflammatory agent.

## 1 Introduction

Inflammation is a defense mechanism of the immune system towards infection or injury. The purpose of inflammation is to eliminate harmful and foreign stimuli and restore tissue structure and physiological function. Inflammation can be classified into two types, which are acute and chronic inflammation (Freire and Van Dyke, 2013; Pahwa et al., 2020). Acute inflammation is an inflammatory response that occurs immediately after injury, lasting only a few days while a response of longer duration is called chronic inflammation. (Kumar et al., 2013). Failure in resolving acute inflammation may lead to chronic inflammation that will contribute to the progression of tissue damage and consequent functional impairments (Kumar et al., 2013).

Nowadays, non-resolving inflammation is becoming a major trigger in various diseases as it plays a significant role in the pathogenesis of atherosclerosis, asthma, cancer, obesity, inflammatory bowel disease, chronic obstructive pulmonary disease, neurodegenerative disease, multiple sclerosis, and osteoarthritis (Nathan and Ding, 2010; Siti et al., 2015; Chow and Chin, 2020). Anti-inflammatory therapies that are currently used are nonsteroidal anti-inflammatory drugs (NSAIDs), glucocorticoids, and disease-modifying agents of rheumatoid diseases (DMARDs) (Tabas and Glass, 2013). However, prolonged use of these drugs is usually accompanied by various side effects (Bhala et al., 2013) such as gastrointestinal bleeding (Moore et al., 2015; Lee Pok et al., 2018), myocardial infarction, heart failure (Schmidt et al., 2016) and kidney injury (Dixit et al., 2010). Therefore, it is important to find a new anti-inflammatory therapy with better efficacy, greater safety and a more economical way to treat inflammation.

**FIGURE 1:**
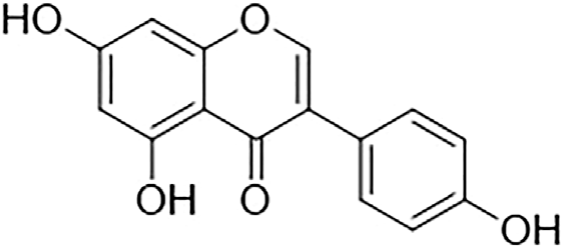
Chemical structure of genistein.

Genistein belongs to the flavonoid family, in the subgroup of isoflavones. It is a phytoestrogen that is mainly derived from legumes such as *Lupinus albus* L. (lupine), *Vicia faba* L (fava bean), *Glycine max* (L.) Merr. (soybeans), *Pueraria lobata* (Willd.) Ohwi (kudzu), and *Psoralea corylifolia* L. (Psoralea). Genistein, chemically known as 5,7-dihydroxy-3-(4-hydroxyphenyl)chromen-4-one, is a naturally occurring chemical constituent with a similar chemical structure to mammalian estrogens. (Kaufman et al., 1997; Dixon and Ferreira, 2002; Verdrengh et al., 2003). It has a molecular formula of C_15_H_10_O_5_ and a molecular weight of 270.241 g/mol. The chemical structure of genistein is shown in Figure 1. Genistein consists of 15 carbons arranged in two aromatic rings (ring A and B) which are connected to another carbon pyran ring (ring C), made up of the 3-phenylchromen-4-one nucleus. The basic carbon skeleton of genistein has a double bond between positions two and three. Also, it has an oxo group at position four of ring C, and three additional hydroxyl groups at positions five and seven of ring A and position four of ring B (Tuli et al., 2019).

Genistein is claimed to exert many beneficial effects on health, such as protection against osteoporosis, reduction in the risk of cardiovascular disease, alleviation of postmenopausal symptoms and anticancer properties (Marini et al., 2007; Si and Liu, 2007; Thangavel et al., 2019; Chen et al., 2020). Apart from that, genistein exerts evident anti-inflammatory properties by affecting granulocytes, monocytes, and lymphocytes that can serve as a novel source of potential phytotherapeutic agents for anti-inflammatory therapies (Dixon and Ferreira, 2002; Verdrengh et al., 2003). However, documentation of anti-inflammatory activities of genistein is yet to be established. Additionally, due to the potential increase in residual activities of defective lysosomal enzymes that would otherwise be subjected to efficient ubiquitination and proteasomal degradation as misfolded proteins, the genistein-mediated reduction of proteasomal activities may have beneficial effects in mucopolysaccharidosis patients’ cells (Pierzynowska et al., 2020). The most current findings from investigations on the use of genistein in various neurodegenerative disease models have described a focus on its autophagy-dependent activity (Pierzynowska et al., 2021). Another activity of genistein is that there are studies to have shown that genistein suppresses epidermal growth factor receptor kinase activity, which is essential for complete expression of genes coding for glycosaminoglycan-producing enzymes. This could be used in anticancer treatments as well as the therapy of several hereditary illnesses related to lysosomal storage diseases (Piotrowska et al., 2006).

Hence, this review is particularly focused on the summarization of the anti-inflammatory properties of genistein linking with the signaling pathways and mediators that are involved in the inflammatory response. Moreover, toxicological investigation of the plant families is also highlighted in this review.

## 2 Methodology

### 2.1 Data Sources and Searches

A wide range of literature searches regarding the anti-inflammatory activity of genistein was conducted using the following databases: PubMed, ScienceDirect, SpringerLink and Scopus. The references of retrieved papers were also searched for additional studies. Search terms were not limited to a specific timeframe, which aimed to collect all studies related to the anti-inflammatory activity of genistein. Search terms were board to ensure all relevant studies were collected. Examples of search terms used were Genistein AND (anti-inflammatory OR anti-inflammation OR “NF-KappaB” OR “NF-*κ*B” OR “NF-*κ*B” OR “iNOS” OR inducible nitric oxide synthase OR pro-inflammatory cytokines OR “ROS” OR reactive oxygen species OR antioxidant OR lipid peroxidation OR prostaglandin OR cyclooxygenase OR “COX” OR 5-lipoxygenase OR “5-LOX” OR platelet-activating factor OR “PAF”). The papers to be included need to fulfill the inclusion criteria as followed: original journals or original research paper related to any anti-inflammatory activity exhibited by genistein compound, genistein used can either be synthetic or an isolated compound but not in crude extract or mixture, published in English, conducted *in vivo* and/or *in vitro* assay. The exclusion criteria for this review: incomplete article and review article.

### 2.2 Data Extraction

The data of the included articles are extracted into a table which includes the information of plant name or source of compound, cell type or subject, assay type, activity and mechanism of action, effective dose or concentration, and the correlated experimental result value. Finally, cytotoxicity data were included.

## 3 Review

### 3.1 Anti-Inflammatory Activity

Genistein, an isoflavone mainly derived from legumes, was shown to has exhibited versatile pharmacological activities such as anti-inflammatory (Ji et al., 2011; Jeong et al., 2014), antioxidant (Yoon and Park, 2014), antiangiogenic (Yu et al., 2012; Cheng et al., 2020), anticancer (Ardito et al., 2017; Ardito et al., 2018), antiproliferative activities (Monti and Sinha, 1994), reduction in neurodegeneration (Pierzynowska et al., 2020; Pierzynowska et al., 2021) and also treatment of certain genetic diseases from the group of lysosomal storage diseases (Piotrowska et al., 2006). However, this study is mainly emphasized the anti-inflammatory activity of genistein to provide a direction in the discovery of potential novel, safe and efficacious natural anti-inflammatory agents in the future. In the past, numerous *in vitro* and *in vivo* studies have been conducted to investigate the anti-inflammatory potential of genistein. Therefore, this review attempts to gather all the findings of published scientific information about the anti-inflammatory activities of genistein to ease future research. Table 1 tabulated with the summarization of the remarkable anti-inflammatory activity of genistein. The mechanisms of anti-inflammatory activities elucidated from genistein are extensively discussed in this review.

**TABLE 1:** Genistein with potent anti-inflammatory effect.

Plant name/Source	Cell type/Subject	Assay type	Mechanism of action	References
Synthetic:Calbiochem Novabiochem	Murine macrophages (J774.2) and bovine aortic endothelial cells (BAEC)	*In vitro*	Dose dependent suppression of 6-oxo-PGF_1α_ from endothelial cells and PGF_2α_ from J774.2 macrophages by genistein (0.05, 0.5, 5 or 50 μg ml^−1^) when compared to LPS-only group	Akarasereenont et al. (1994)
			Dose dependent suppression of LPS-induced nitrite accumulation in J774.2 macrophages by genistein (0.05, 0.5, 5 or 50 μg ml^−1^) as compared with LPS-only group	
			Genistein (50 μg ml^−1^) significantly suppressed COX-2 protein which induced by LPS in both BAEC and J774.2 macrophages	
Synthetic:Sigma-Aldrich	HAEC (human aortic EC)	*In vitro*	Concentration-dependent suppression of monocyte adhesion, IL-8 and MCP-1 production in HAEC cultured with high glucose (25 μmol/L) by genistein (0.01, 0.1, 1 and 10 μmol/L), with an effective inhibition at concentration as low as 1 μmol/L (*p* < 0.05)	Babu et al. (2012)
	Five-week-old male diabetic mice	*In vivo*	8 weeks of dietary intake of 0.1% genistein effectively reduced serum concentration of MCP-1/JE, KC, ICAM-1 and VCAM-1 and increased IL-10 concentrations in *db/db* mice (*p* < 0.05)	
Synthetic:Sigma-Aldrich	C57BL/6 male mice (seven weeks-old)	*In vivo*	Significant inhibition of COX-2 (*p* < 0.01) and ICAM-1 (*p* < 0.05) in inflamed region of periodontitis mice with intraperitoneal administration at 20 mg/kg body weight of genistein for 3 weeks	Bhattarai et al. (2017)
	RAW 264.7 macrophages and hGFs	*In vitro*	In LPS-stimulated RAW 264.7 macrophages, pretreatment of genistein (0–70 µM) suppressed NOS2, COX-2, and TNF-α dose-dependently. Genistein also attenuated COX-2 and ICAM-1 levels which induced by LPS in hGFs, but no effect on TNF-α	
	hGFs		Genistein effectively restored the LPS-induced mitochondrial activity and attenuated the cellular ROS level (*p* < 0.05) compared to LPS treatment alone in hGFs	
	RAW264.7 cells	*In vitro*	Pretreatment of genistein (5, 10, 20, 30 or 50 μM) significantly inhibited *Prevotella intermedia* LPS-induced production of NO and IL-6, and induction of iNOS protein in a dose-dependent manner	Choi et al. (2016)
Synthetic:LC Laboratories			Downregulation of *Prevotella intermedia* LPS-induced iNOS and IL-6 mRNA expression by pretreatment of genistein (5, 10, 20, 30 or 50 μM) in a dose-dependent manner	
Synthetic:Indofine Chemical Co.	Female C57Bl/6 mice 8–12 weeks old	*In vivo*	Suppression of IFN-*γ*, IL-12, and TNF-α cytokines and upregulation of IL-10 production in the brain from mice immunized with 100 μg MOG_35–55_ peptide with genistein 200 mg/kg	De Paula et al. (2008)
			Downregulation of IFN-*γ*, IL-10 and TNF-α cytokines on the splenocyte supernatants from mice immunized with 100 μg MOG_35–55_ peptide with genistein 200 mg/kg	
Synthetic:Sigma-Aldrich	^14^C-oleate labeled *E. coli*	*In vitro*	Genistein showed significant inhibitory effects against all sPLA_2_ enzymes of inflammatory exudates and snake venoms in a concentration-dependent manner with IC_50_ values from 5.75 to 11.75 μM. (sPLA_2_ inhibitor)	Dharmappa et al. (2010)
			
Synthetic:Sigma-Aldrich	MoDCs	*In vitro*	Pretreatment of genistein (6.25–200 μM) significantly and dose-dependently inhibited LPS-induced up-regulation of IL-6 in MoDCs with IC_50_ value of 52.07 μM	Dijsselbloem et al. (2007)
	Human embryonic kidney (HEK)293T cells	*In vivo*	Genistein (200 μM) significantly suppressed IL-6 transcription, NF-*κ*B DNA binding (p65-p50 heterodimers and p50 homodimers) and p65 nuclear localization which induced by LPS in MoDCs, while up-regulated protein levels of p53	
	BMDCs from p53^−/−^ and p53^+/+^ mice		Genistein (50, 100 and 200 μM) significantly and dose-dependently suppressed LPS-induced NF-*κ*B-dependent promoter activity (*p* < 0.01) in HEK293T cells as compared to untreated cells	
			Pretreatment of genistein (200 μM) remarkably suppressed both LPS-stimulated IL-6 mRNA levels and p65 nuclear abundance in the majority of p53^+/+^ BMDCs, but no impact in p53^−/−^ BMDCs	
	Murine BV2 microglial cell line and primary microglial culture	*In vitro*	Genistein (5, 10 or 20 μM) showed significant inhibitory effects against LPS-induced up-regulation of iNOS, COX-2, TNF-α, IL-1β and IL-6 in a dose-dependent manner	Du et al. (2018)
Synthetic:Tauto Biotech			Genistein (10 μM) remarkably inhibited LPS-induced activation of mitogen-activated protein kinase (MAPK) and NF-κB with attendant suppression in the phosphorylation of JNK (*p* < 0.05), p38 (*p* < 0.001), ERK (*p* < 0.001) and IκB (*p* < 0.05) in BV2 microglial cells as compared to LPS-only group	
			Upregulation of GPER gene and protein expression by genistein (10 μM) which involved in anti-inflammation	
Synthetic:Sigma-Aldrich	RAW 264.7 cells	*In vitro*	Genistein (20, 40, 60, 80 or 100 μM) significantly and dose-dependently suppressed (*p* < 0.05) nitrite accumulation as compared to LPS-treated control. Genistein with IC_50_ of 50 µM had the strongest inhibitory effect (100 μM, 67.7%) among the isoflavones	Sheu et al. (2001)
			Dose-dependent inhibition of iNOS activity by genistein (25, 50 or 100 µM), with a significant inhibition (36.5%) at a concentration of 100 µM (*p* < 0.05), which is higher than both daidzein (26.7%) and glycitein (19.9%)	
			Downregulation of LPS-induced iNOS protein expression by treatment of genistein (25, 50 or 100 μM) in a dose-dependent manner, with a significant suppression (89%, *p* < 0.05) at a concentration of 100 µM	
			Genistein exhibited significant inhibitory effect (66.4%, *p* < 0.05) against LPS-induced iNOS mRNA expression superior to that of daidzein (57.8%) and glycitein (57.2%) at the doses of 100 µM	
Synthetic:Sigma-Aldrich	Male Wistar rats	*In vivo*	Pretreatment of genistein (5 mg/kg/day) significantly inhibited _D_-GalN induced up-regulation of TNF-α and IL-1β levels, and expression of iNOS and COX-2 (*p* < 0.05)	Ganai et al. (2015)
			Significant suppression of _D_-GalN induced NF-κB, MAPK (p-38, ERK 1/2) and IKKα/β expression with pretreatment of 5 mg/kg genistein (*p* < 0.05) as compared with _D_-GalN-induced group with the absence of genistein	
Synthetic:Sigma-Aldrich	RAW 264.7 cell	*In vitro*	Genistein significantly and dose dependently inhibited IFN-*γ* plus LPS-induced nitric oxide production (5–100 μM, *p* < 0.05) and tumour necrosis factor secretion (50–100 μM, *p* < 0.05) in RAW 264·7 macrophages with IC_50_ of 57.9 and 52.9 μM respectively. Also, genistein has a superior inhibitory effect than daidzein, where higher concentration of daidzein was necessary to significantly inhibited nitric oxide production (50–100 μM, *p* < 0.05) in LPS-induced macrophages	Gottstein et al. (2003)
			
Synthetic:Extrasynthese	Murine J774 macrophages	*In vitro*	Treatment of genistein (100 µM) showed inhibitory effect (*p* < 0.01) against LPS-induced PGE_2_ production (89.8 ± 0.8%), COX-2 mRNA (54.9 ± 5.8%) and protein expression (40.8 ± 7.0%)	Hämäläinen et al. (2011)
			
Synthetic:Extrasynthese	Murine J774 macrophages	*In vitro*	Genistein (10–100 μM) significantly and dose-dependently suppressed LPS-induced NO production in J774 macrophages with IC_50_ of 30 µM. Its inhibitory effect at dose of 100 μM (97.4%) was comparable with that of the positive controls, NOS inhibitor L-NIO (1 mM) and a selective iNOS inhibitor 1400W (1 mM) (>90%)	Hämäläinen et al. (2007)
			At concentration of 100 μM, genistein significantly inhibited both LPS-induced iNOS protein and mRNA expression in J774 cells (*p* < 0.01)	
			Genistein (100 μM) interfered LPS-induced activation of NF-κB (57% inhibition, *p* < 0.01) and STAT-1 (32% inhibition, *p* < 0.01) in J774 cells as compared to LPS-treated alone cell	
Synthetic:Sigma-Aldrich	Human umbilical vein endothelial cell (ECV-304)	*In vitro*	Pretreatment of genistein (10, 50 and 100 µM) dose-dependently inhibited the generation of IL-6, ICAM-1 and ROS induced by HCY in ECV-304 cells	Han et al. (2015)
			Pretreatment of genistein (10, 50 and 100 μM) exhibited prominent inhibitory effect on the expression of NF-κB p65 protein induced by homocysteine (HCY) in a dose-dependent manner. Result highlighted that 100 μM of genistein almost completely abolished the nuclear translocation of NF-κB in ECV-304 cells as compared with HCY-only group (*p* < 0.01), reaching level comparable to the control group (*p* > 0.05)	
	Normal human chondrocytes	*In vitro*	Treatment of genistein (100 µM) effectively inhibited LPS-induced upregulation of COX-2 protein level (*p* < 0.05) as compared with LPS-only group, but no effect on COX-1 protein level	Hooshmand et al. (2007)
Synthetic:Sigma-Aldrich			At dose of 50 μM, genistein significantly inhibited LPS-induced NO production in cell culture supernatants (*p* < 0.05)	
			Treatment of genistein at doses of 50 and 100 µM showed inhibitory effect against LPS-induced IL-1β production by 36.4 and 48% respectively	
Synthetic:Sigma-Aldrich	The rat gland pheochromocytoma (PC12) (BCRC 60048) cell line	*In vitro*	Genistein (2, 5 and 10 µM) significantly and dose-dependently suppressed DG-induced intracellular ROS levels (*p* < 0.05) in PC12 cells when compared to DG-only group	Hsieh et al. (2011)
			Genistein (2–10 μM) completely inhibited DG-induced increase in the binding activity of NF-κB (*p* < 0.05) when compared to DG-only group	
			Treatment of genistein at concentration of 0.5, 2, 5 and 10 µM completely restored the DG-induced suppression of IκB-α protein expression (*p* < 0.05) in PC12 cells when compared to DG-only group	
Synthetic:Sigma-Aldrich	Highly aggressive proliferating immortalized (HAPI) microglial cells	*In vitro*	Pretreatment of genistein at concentration of 0.01, 0.1 and 1 μM for 1 h significantly and dose-dependently suppressed LPS-induced NO production (*p* < 0.05) compared to cell treated with LPS alone, which was similar to that of the positive control, estradiol (0.0001–0.1 μM)	Jantaratnotai et al. (2013)
			Genistein (1 μM) significantly suppressed LPS-induced increase in iNOS mRNA expression (*p* < 0.05) which was comparable with that of the positive control, 0.1 μM of estradiol	
			Pretreatment of genistein (1 µM) effectively reduced the LPS-induced upregulation of iNOS, IRF-1 and pSTAT1 protein expression, and MCP-1 and IL-6 mRNA expression in HAPI cells by half (*p* < 0.05) which was comparable with that of the positive control, 0.1 μM of estradiol	
	BV2 microglia	*In vitro*	Genistein (25 and 50 µM) significantly and concentration-dependently inhibited LPS-induced NO and PGE_2_ production (*p* < 0.05), iNOS and COX-2 mRNA and protein expression in BV2 microglia	Jeong et al. (2014)
Synthetic:Sigma-Aldrich			Pretreatment of genistein (25 and 50 µM) effectively and concentration-dependently suppressed the LPS-induced TNF-α and IL-1β production (*p* < 0.05), and gene expression by reduction in their mRNA and protein level	
			At concentration of 50 μM, pretreatment with genistein significantly inhibited LPS-induced ROS levels and NF-κB p65 nuclear translocation	
			Pretreatment of genistein (25 and 50 μM) concentration-dependently inhibited LPS-induced increase in the nuclear NF-κB p65 levels and completely restored the degradation of cytosolic IκB-α protein	
Synthetic: Cayman Chemical Co.	RAW 264.7 mouse macrophage cells	*In vitro*	Genistein (0.1, 1, 5, or 10 μM) dose-dependently inhibited LPS-induced TNF-α (*p* < 0.05, at dose ≥ 1 μM) and IL-6 (*p* < 0.05, at dose ≥ 5 μM) mRNA levels in macrophages as compared to LPS-only group. Also, the suppressive effect exhibited by 10 μM of genistein was similar to that with the positive control, AMPK agonist AICAR (1 mM)	Ji et al. (2012)
			Pretreatment of genistein (1, 5, or 10 μM) dose-dependently and time-dependently inhibited LPS-induced increase in the nuclear NF-κB p65 protein, and phosphorylation of IKKα/β in macrophages. Genistein also restored the degradation of cytosolic IκB-α protein and decrease in AMPK phosphorylation in a dose-dependent and time-dependent manner	
Synthetic: Cayman Chemical Co.	Male Sprague–Dawley rats (six-weeks-old)	*In vivo*	Intragastrical administration of genistein (4 and 8 mg/kg/day) for 12 weeks significantly and dose-dependently inhibited HFD induced up-regulation of TNF-α and IL-6 levels, and their mRNA expression in serum and liver of NASH rats	Ji et al. (2011)
			Significant inhibition (*p* < 0.05) of HFD-induced phosphorylation of JNK with genistein 4 and 8 mg/kg, while no significant difference (*p* > 0.05) in p38 and ERK 1/2 as compared to HFD-only group	
			Pretreatment of genistein (4 and 8 mg/kg/day) significantly and dose-dependently inhibited HFD-induced nuclear NF-κB p65 and cytoplasmic phosphorylated IκB-*α* expression (*p* < 0.05) as compared to HFD-only group. Also, genistein restored the degradation of cytoplasmic IκB-*α* protein (*p* < 0.05 or *p* < 0.01)	
*Pueraria lobata*	RAW 264.7 murine macrophages	*In vitro*	Genistein isolated from *P. lobata* roots showed significant inhibitory effects against LPS-induced NO production with IC_50_ value of 8.08 ± 1.17 µM, which was comparable to that of the positive control, AMT (IC_50_ of 0.004 ± 0.00 µM)	Jin et al. (2012)
Synthetic	Female 8-week-old BALB/c mice	*In vivo*	Oral administration of 3 ml genistein (3.33 mg/ml) remarkably inhibited IL-1β, IL-6, and PGE_2_ secretion in both peritoneal exudate cell and peritoneal exudate fluid for BALB/c mice, which was comparable with that of the positive control, ammonium pyrrolidinedithiocarbamate (PDTC) 100 mg/kg	Kao et al. (2007)
LC Laboratories			Genistein significantly suppressed NaNO_2_ secretion in peritoneal exudate cell which was comparable with PDTC 100 mg/kg, but no significant effect in peritoneal exudate fluid	
Synthetic:Sigma-Aldrich	Human leukemic mast cell (HMC)-1 line	*In vitro*	Pretreatment of genistein (12.5, 25 and 50 µM) effectively and concentration-dependently attenuated the PMA/A23187-induced IL-1β and IL-6 gene expression in HMC-1, but no effect in TNF-α. Genistein also robustly reduced PMA/A23187-induced IL-6 production in HMC-1 (*p* < 0.01)	Kim et al. (2014)
			Pretreatment of genistein (50 µM) showed inhibitory effect against PMA/A23187-induced phosphorylation of ERK1/2 in mast cells	
Synthetic:Sigma-Aldrich	Specific pathogen free (SPF) male Sprague–Dawley (SD) rats	*In vivo*	Treatment of genistein (2 and 4 mg/kg/day) for 10 days effectively and dose-dependently inhibited age-related increase in ROS and ONOO- level	Kim et al. (2011)
	Rat endothelial cell lines, YPEN-1	*In vitro*	Genistein (2 and 4 mg/kg/day) effectively and dose-dependently inhibited (*p* < 0.05) age-related phosphorylation of cytosolic IKKα/β and IκB-α as compared to old, untreated group. Also, genistein restored the degradation of cytoplasmic IκB-α protein (*p* < 0.05)	
			Genistein (2 and 4 mg/kg/day) significantly and dose-dependently inhibited (*p* < 0.05 or *p* < 0.01) NF-κB nuclear translocation of p65 and p50, and phosphorylation of nuclear p65 subunit (Ser 536) in aged rats as compared to untreated group	
			Treatment of genistein (2 and 4 mg/kg/day) for 10 days effectively and dose-dependently suppressed age-related increase in renal COX-2, 5-LOX and MCP-1 levels (*p* < 0.01)	
			Genistein (1 and 5 µM) showed significant and dose-dependent inhibition against Ang II-induced production of ROS, renal COX-2 and MCP-1 in YPEN-1 cells (*p* < 0.01)	
			Genistein (1 and 5 µM) significantly inhibited Ang II-induced phosphorylation of cytosolic IKKα/β and IκB-α (*p* < 0.01). Also, genistein restored the degradation of cytoplasmic IκB-α protein (*p* < 0.01)	
			Genistein (1 and 5 µM) effectively inhibited NF-κB nuclear translocation of p65 and p50, and phosphorylation of nuclear p65 subunit (Ser 536) in YPEN-1 cells (*p* < 0.01)	
Synthetic:Longpu Technology	MH7A cells	*In vitro*	Concentration-dependent inhibition (*p* < 0.05 or *p* < 0.01) on TNF-α-induced proinflammatory cytokine production such as IL-1β, IL-6, and IL-8 with genistein (5, 10, 20 µM) in MH7A cells as compared to TNF-α-only group. Results showed that genistein at concentration of 20 µM possessed stronger inhibitory effect than NAC (10 mM), PI3K inhibitor LY294002 (20 µM) and AICAR (1 mM) in TNF-α-stimulated MH7A cells	Li et al. (2014)
			Promotion of AMPK activation and significant inhibition of TNF-α-induced NF-κB p65 nuclear translocation, IKK/IκB/NF-κB pathway and ROS/Akt/NF-κB pathway with genistein (20 µM)	
Synthetic:Sigma-Aldrich	Human chondrocytes	*In vitro*	Genistein 10 µM significantly inhibited nitrite and ROS production in IL-1β-induced OA chondrocytes as compared with IL-1β only group (*p* < 0.01). Also, genistein significantly reduced the expression of NOS2 and COX-2 in chondrocytes (*p* < 0.01)	Liu et al. (2019)
	Institute of Cancer Research (ICR) mice (4 weeks, 20–22 g)	*In vivo*	Genistein 10 mg/kg significantly reduced the protein expression of NF-κB p65 in the cortex of CSD-treated mice (*p* < 0.05), which was comparable with that of the positive control, modafinil (MOD) 100 mg/kg	Lu et al. (2020)
Synthetic			Genistein (10, 20 and 40 mg/kg) significantly downregulated CSD-induced iNOS and COX-2 protein expression in the cortex (*p* < 0.05, *p* < 0.01 or *p* < 0.001), which was comparable with that of the positive control, modafinil (MOD) 100 mg/kg	
Shenggong Biological Engineering Co. Ltd.			In the hippocampus of CSD-treated mice, genistein (10, 20 and 40 mg/kg) showed significant inhibitory effects against NF-κB, iNOS, and COX-2 protein expression, which was comparable with that of the positive control, modafinil (MOD) 100 mg/kg. However, genistein 10 mg/kg had no inhibitory effect against protein expression of iNOS.	
			Genistein (10, 20 and 40 mg/kg) significantly suppressed the level of TNF-α, IL-6 and IL-1β in the serum of CSD mice. Also, treatment of positive control, modafinil (MOD) 100 mg/kg markedly inhibited IL-6 and IL-1β (*p* < 0.001) but no significant difference in level of TNF-α in the serum of CSD-treated mice	
Synthetic:Sigma-Aldrich	Cortical primary astrocyte cultures	*In vitro*	Pretreatment of genistein (50 µM) significantly inhibited NF-κB nuclear translocation of p65 and NF-κB DNA binding in the hemolysate-induced astrocytes as compared to hemolysate stimuli without genistein	Lu et al. (2009)
			Pretreatment of genistein (5, 10 and 50 µM) significantly and concentration-dependently inhibited hemolysate-induced iNOS and COX-2 mRNA protein expression in astrocytes (*p* < 0.05)	
Synthetic:Sigma-Aldrich	C6 cells (rat glioma cell line)	*In vitro*	Genistein (50 µM) significantly suppressed the level of IL-1β and TNF-α induced by A*β*25-35 in C6 cells (*p* < 0.05)	Ma et al. (2015)
			Also, genistein restored the degradation of both IκB-*α* protein and mRNA expression in C6 cells damaged by A*β*25-35 (*p* < 0.05)	
	Peripheral blood mononuclear cells (PBMCs)	*In vitro*	Genistein (10 and 25 µM) significantly suppressed IFN-*γ* production induced by IL-12/IL-18 in cell culture supernatants from PBMCs (*p* = 0.0023)	Mace et al. (2019)
Synthetic:LC Laboratories			Genistein (25 µM) significantly reduced IFN-γ intracellular staining in CD3^−^NK^Dim^ (open) and CD3^−^CD56^Bright^ (shaded) NK cells (*p* = 0.0153 and *p* = 0.0147 respectively)	
			Genistein (25 µM) decreased IL-12/18-induced IL-18Rα expression on CD56 ^+^ NK cells (*p* < 0.01), but no impact on the expression of IL-12Rβ1	
Purified from defatted soy flour	Soy lipoxygenase and human PMNL 5-lipoxygenase	*In vitro*	Genistein inhibited soy lipoxygenase and human PMNL 5-lipoxygenase with IC_50_ values of 107 and 125 μM, respectively	Mahesha et al. (2007)
Synthetic:Cayman Chemical	Male albino Wistar rats	*In vivo*	Genistein (50 or 100 mg/kg) significantly reduced LPS-induced upregulation of NF-κB, IL-6, TNFα, TLR4, GFAP, iNOS and COX-2 in hippocampal level, which was comparable to that of the positive control, dexamethasone (0.2 mg/kg)	Mirahmadi et al. (2017)
Synthetic:Sigma-Aldrich	Male Hartley guinea pigs	*In vivo*	Significant inhibition of TNBS-induced myeloperoxidase activity (index of neutrophil infiltration) with genistein 0.1 mg/kg	Sadowska-Krowicka et al. (1998)
	RAW264.7 cells	*In vitro*	Significant inhibition of TNBS-induced increase in nitrite production with genistein 0.1 mg/kg	
			Reduction of positive staining for iNOS and nitrotyrosine associated with TNBS administration and improved mucosal morphology by genistein 0.1 mg/kg	
			Genistein (10 and 100 kg/ml) and iNOS inhibitor, NIL (5 mM) markedly inhibited LPS-induced nitrite production when compared with LPS-treated cells (*p* < 0.05)	
Synthetic:Pharmaceutical Research Institute	Spontaneously immortalized human keratinocytes (HaCaT cell line)	*In vitro*	Pretreatment of genistein (100 µM) for 2 h inhibited TNF-α-induced NF-κB nuclear translocation of p65 in keratinocytes	Smolinska et al. (2018)
			Genistein (100 µM) significantly suppressed expression of intracellular ROS which induced by TNF-α and LPS in HaCaT cells (*p* ≤ 0.05)	
			Genistein significantly inhibited levels of IL-8, IL-20, and CCL2 which induced by ACT, TNF-α and LPS in HaCaT cells (*p* ≤ 0.05 or *p* ≤ 0.001), except for IL-20 induced by TNF-α and LPS. Most of the inhibitory effect of genistein is stronger than the positive control, methotrexate (1 µM) except for IL-8 induced by TNF-α (3 times potent than genistein)	
Synthetic:Bioword	Female mice (*Mus musculus*) age 2,3 months, weigh 20–30 g	*In vivo*	Genistein (1.04 and 1.3 mg/day) significantly reduced the expression of TNF-α and IL-6 in mice model of endometriosis (*p* < 0.05), which was comparable with the positive control, leuprolide acetate (0.00975 mg/5 days)	Sutrisno et al. (2018)
Synthetic	Primary endometriosis cells	*In vitro*	Genistein (5 until 50 μM) significantly reduced the level of TNF-α and IL-6 in supernatant cells as compared with control group in all duration of treatment (6, 24 and 48 h) (*p* < 0.05)	Sutrisno et al. (2014)
			Significant downregulation of the level of IL-1β was shown in the culture of endometriosis cells with genistein as compared with control group for 6, 24 and 48 h incubation period (20–50 μM, 5–50 μM and 10–50 μM respectively) (*p* < 0.05)	
Synthetic	Female mice (*Mus musculus*) age 2–3 months, weigh 20–30 g	*In vivo*	Genistein (0.78 and 1.3 mg/day) significantly decreased the expressions of NF-κB and COX-2 in mice model of endometriosis (*p* < 0.05). Both positive controls, leuprolide acetate (0.00975 mg) and dienogest (0.0052 mg) decreased the expression of NF-κB and COX-2, but no significant difference as compared with endometriosis group (*p* > 0.05)	Sutrisno et al. (2017)
Bioword			Also, genistein (0.78, 1.04 and 1.3 mg/day) portrayed significant inhibitory effect on the expression of PGE (*p* < 0.05) in comparison to the endometriosis group, which was equivalent to that of dienogest	
Synthetic:Tokyo Chemical Industries	Female mice (*Mus musculus*) age 2,3 months, weigh 20–30 g	*In vivo*	Genistein (50–500 mg/day) significantly suppressed EM-induced increase in TNF-α, IL-1β, IL-6 and IL-9 level, and NF-κB expressions in peritoneal fluid of mice model endometriosis (*p* < 0.05)	Sutrisno et al. (2015)
Synthetic:Sigma-Aldrich	PCa cell lines and primary prostatic epithelial cells	*In vitro*	Genistein (10 μM) significantly suppressed COX-2 mRNA expression in PCa cell lines and primary prostatic epithelial cells	Swami et al. (2009)
			Genistein significantly decreased PG receptor (*EP4 and FP*) mRNA expression in LNCaP (*p* < 0.001 and *p* < 0.05 respectively), but no significant effect in PC-3. Also, genistein effectively reduced *EP4* mRNA levels in primary prostatic epithelial cells (E-PZ-1, -2 and -3, and E-CA-1 and -3)	
			Genistein (10 μM) significantly decreased PGE_2_ secretion in LNCaP cells, PC-3 cells and primary epithelial cell cultures	
	Human CF and non-CF bronchial tissue	*In vitro*	Genistein (20 μM, 16 h) significantly upregulated cytoplasmic IκB-*α* protein in CF HBG cells	Tabary et al. (1999)
Synthetic:Sigma-Aldrich			Genistein (100 μM) significantly inhibited LPS-induced NF-*κ*B activity in both CF and non-CF HBG cells	
			Treatment of genistein (20 and 100 μM) significantly inhibited IL-8 production in both CF and non-CF HBG cells in a dose- and time-dependent manner (*p* < 0.001)	
Synthetic:Wako Pure Chemical Industries, Ltd.	Seven-week-old male Wistar rats	*In vivo*	Genistein (50 and 100 mg/kg/day) significantly and dose-dependently suppressed increase in MPO activity, TBARS level, TNF-α and IL-8 (CINC-1) concentrations in the gastric mucosa of WIR-stressed rats	Takekawa et al. (2006)
Synthetic	C57BL/6 male mice at 10–12 weeks of age (20–25 g)	*In vivo*	Genistein (0.2, 1 and 5 mg/kg) significantly inhibited the diabetes-induced cutaneous O_2_^·^−and nitrotyrosine production, while increased the nitrite level	Tie et al. (2013)
			Genistein (0.2, 1 and 5 mg/kg) effectively inhibited cutaneous iNOS activity in diabetic mice, while genistein 5 mg/kg increased cNOS activity	
Synthetic	C57BL/6J male mice, weighing 20–25 g	*In vivo*	Genistein 3 and 6 mg/kg significantly inhibited the diabetes-induced increase in proinflammatory cytokine level such as TNFα, IL1β and IL6 in sciatic nerve (*p* < 0.05 or *p* < 0.01). Also, genistein 3 mg/kg completely reverted the increase in reactive oxygen species level (*p* < 0.01)	Valsecchi et al. (2011)
			Genistein 3 mg/kg significantly reverted the decrease in eNOS content in thoracic aorta of diabetic mice (*p* < 0.001). Also, genistein effectively abolished the increase in iNOS content and SOD activity (*p* < 0.01 and *p* < 0.05 respectively)	
			In brain of diabetic mice, genistein 3 and 6 mg/kg significantly attenuated increase in oxidative marker levels such as reactive oxygen species and MDA (*p* < 0.05). Also, genistein 3 and 6 mg/kg effectively suppressed the activities of antioxidant defense enzymes such as GR (*p* < 0.05) but no significant impacts on SOD, GPx and catalase	
			In liver of diabetic mice, genistein 3 and 6 mg/kg significantly suppressed increase in reactive oxygen species level, while only genistein 6 mg/kg attenuated MDA increase (*p* < 0.05). Genistein 3 and 6 mg/kg completely restored the activity of GPx in liver (*p* < 0.05) but no effect on catalase activity. Genistein 6 mg/kg further increased the hepatic GR activity (*p* < 0.05) but genistein 3 mg/kg did not modify this increase	
Synthetic: Nanjing Zelang Medical Technology Company	Male BALB/c mice (7–8 weeks old)	*In vivo*	Genistein 0.5 and 2% effectively attenuated the cytokine level and mRNA expression in IMQ-induced mouse skin, including IL-1β, IL-6, TNF-α, IL-17 and IL-23. Also, genistein significantly suppressed chemokine CCL2 mRNA level and cytokine MCP1 level in psoriasis-like lesions. Genistein was proved to have a superior inhibition effect than the positive control, Daivonex (calcipotriol ointment)	Wang et al. (2019)
	Human keratinocyte HaCaT cells	*In vitro*	Genistein (50 and 100 μM) significantly and dose-dependently suppressed TNF-α-induced mRNA expression of IL-1β, IL-6, IL-8, IL-23, TNF-α, VEGFA and CCL2 in HaCaT cells	
			Genistein (100 μM) significantly abolished the increase in IL-1β, IL-6, IL-8, IL-23, TNF-α, VEGFA and MCP1 level in TNF-α-treated HaCaT cells	
			Genistein (50 and 100 μM) significantly and concentration-dependently suppressed TNF-α-induced phosphorylation of IκB-α (*p* < 0.01 and *p* < 0.001 respectively) as compared to TNF-α-only group	
			Genistein (100 μM) significantly attenuated TNF-α-induced increase in NF-κB level (*p* < 0.01) in HaCaT cells as compared to TNF-α-only group	
*Glycine max* L. Merr	RAW 264.7 cell	*In vitro*	At concentration of 40 μg/ml, genistein significantly inhibited activity of inflammatory mediators such as PGE_2_, TNF-α, and IL-1β	Widowati et al. (2019)
Synthetic:Sigma-Aldrich	Vascular Smooth Muscle Cells (VSMCs)	*In vitro*	Genistein (10^−4^, 10^−5^ and 10^−6^ M) significantly and concentration-dependently suppressed Ang II-induced protein expressions of *p*-ERK1/2, p-p38 and NF-κB in VSMCs, while restored the downregulations of ERβ and PPARγ	Xu et al. (2019)
Synthetic:Sigma-Aldrich	Female Wistar rats (weighing 180–220 g, about 10 weeks old)	*In vivo*	Treatment with genistein (1 mg/kg/day) effectively suppressed NF-κB and IL-1β protein levels in the pancreas of ovariectomized diabetic rat, while completely restored the downregulation of SIRT1 protein levels	Yousefi et al. (2017)
Synthetic:Sigma-Aldrich	BV-2 cell	*In vitro*	Pretreatment with genistein (50 μM) significantly suppressed Aβ25-35-induced increase in RNA and protein expression of IL-6 while restored the decrease in IL-10	Yu et al. (2013)
			Pretreatment with genistein (50 μM) effectively abolished the elevation of TGF-β mRNA level induced by Aβ25-35 in BV-2 cell, but no significant effects on protein expression	
				
*Sophora japonica*	Murine early B cell line Y16, BAF/BO3 cell line, human erythroleukemia TF-1, human melanoma A375.S2, murine fibrosarcoma WEHI-164 and hybridoma MH60/BSF-2	*In vitro*	Genistein showed significant inhibitory effects against IL-5, IL-3, IL-6 and GM-CSF in a concentration-dependent manner with IC_50_ values of 19.4, 28.4, 13.3 and 59.8 μM respectively. However, it showed no inhibitory effects on both IL-1β and TNF-α	Yun et al. (2000)
Synthetic	Female BALB/C mice	*In vivo*	Treatment of genistein (600 mg/kg) remarkably suppressed DSS-induced colonic production of IL-1β and IFN-γ (*p* < 0.05) but no significant effects on IL-6, IL-10, IL-17 and TNF-α	Zhang et al. (2017)
	Human epithelial Caco-2 cells	*In vitro*	Genistein decreased DSS-induced nuclear NF-κB p65 abundance and suppressed TLR4 expression in Caco-2 cells (*p* < 0.05)	
Synthetic:Sigma-Aldrich	Male Wistar rats	*In vivo*	Genistein (2 and 20 mg/kg/d) significantly and dose-dependently suppressed LPS-induced production of TNF-α, IL-1β, and IL-6 in both liver slice culture supernatant and serum (*p* < 0.05)	Zhao et al. (2006)
	Cultured rat liver slices	*In vitro*	Genistein (0.186–370 μM) robustly and concentration-dependently inhibited LPS-induced TNF-α production in liver slice culture (*p* < 0.05) as compared with positive control, treatment with vehicle (5% DMSO)	
Synthetic:Sigma-Aldrich	C6 cells (rat glioma cells)	*In vitro*	Genistein (50 μM) effectively abolished the elevation of IL- 6, iNOS and COX-2 in Aβ25-35-treated C6 cells (*p* < 0.05) but no significant effect on the IL-4 level	Zhao et al. (2014)
			Genistein (50 μM) remarkably suppressed Aβ25-35-induced increase in NF-κB p65 protein expression in C6 cells (*p* < 0.05) but no significant effect on the mRNA expression (*p* = 0.343)	
SyntheticSigma-Aldrich	BV-2 cell line	*In vitro*	Genistein (50 μM) effectively abolished the elevation in both mRNA and protein expression of proinflammatory cytokines, such as IL-1β and iNOS in Aβ25-35-treated BV-2 cells (*p* < 0.05) while restored the decrease in expression of IL-10	Zhou et al. (2014)
			Genistein (50 μM) remarkably suppressed mRNA and protein expression of NF-κB p65, NF-κB p50 and toll-like receptor 4 (TLR4) in BV-2 microglia (*p* < 0.05) when compared to Aβ25-35-only group. However, there was no statistical significance found in mRNA expression of NF-κB p50 with treatment of genistein (*p* > 0.05) when compared with other groups	
				
Synthetic:Shanghai Ronghe Medical Science and Technology Development Co.	MLE-12 cells	*In vitro*	Genistein (1 and 10 μM) remarkably and concentration-dependently suppressed LPS-induced increase in TNF-α, IL-1β, IL-6, and KC in MLE-12 cells (*p* < 0.001). Its suppressive effects at a dose of 10 μM was comparable with that of the positive control, FK866 (PBEF inhibitor) (10 nm)	Zhu et al. (2020)
			Genistein (10 μM) effectively abolished the elevation in both expressions of PBEF (*p* < 0.001) and nuclear p65 (*p* < 0.001) in LPS-treated MLE-12 cells, while restored the decrease in expression of cytoplasm p65 (*p* < 0.001) as compared to LPS-only group. Its activity was comparable with that of the positive control, FK866 at concentration of 10 nm	

#### 3.1.1 Nuclear Factor Kappa-B Inhibition

Nuclear factor-κB (NF-κB) represents a family of inducible transcription factors that play critical roles in various processes of the immune and inflammatory responses. (Oeckinghaus and Ghosh, 2009; Sun et al., 2013; Liu et al., 2017). Activation of NF-κB induces the transcription of several genes such as chemokines, cytokines and adhesion molecules in various innate immune cells, thus directly regulates inflammatory response. Apart from that, NF-κB involves indirectly in the inflammatory process by promoting the differentiation of inflammatory T cells and initiating the regulation of cell proliferation, apoptosis, morphogenesis and differentiation (Liu et al., 2017; Choy et al., 2019). Therefore, a compound with an inhibitory effect on NF-κB activation may be the potential candidate of a new anti-inflammatory agent.

There was a study carried out by Hämäläinen et al. (2007) to determine the effects of flavonoids on activation of NF-κB and signal transducer and activator of transcription 1 (STAT-1) by analyzing the nuclear translocation. In the study, 100 μM of genistein significantly interfered lipopolysaccharides (LPS)-induced activation of NF-κB (57% inhibition, *p* < 0.01) and STAT-1 (32% inhibition, *p* < 0.01) in J774 cells as compared to LPS-treated alone cell (Hämäläinen et al., 2007). Also, Lu et al. (2009) showed the inhibitory effect of genistein pretreatment (50 µM) on the NF-*κ*B nuclear translocation of p65 and DNA binding in the hemolysate-induced astrocytes as compared to hemolysate stimuli without genistein (Lu et al., 2009). In another account, Hsieh et al. (2011) explored the influence of genistein on D-galactose (DG)-induced oxidative damage in PC12 cells. The result demonstrated that genistein robustly inhibits DG-induced increase in the binding activity of NF-*κ*B (2–10 μM, *p* < 0.05), as well as restored the suppression of I*κ*B-*α* protein expression (0.5–10 μM, *p* < 0.05) in PC12 cells when compared to DG-only group (Hsieh et al., 2011).

Aside from that, Ji et al. (2012) proposed that pretreatment of genistein (1, 5, or 10 μM) dose-dependently and time-dependently inhibited LPS-induced increase in the nuclear NF-*κ*B p65 protein and phosphorylation of IKK*α*/*β* in macrophages. In addition, genistein restored the degradation of cytosolic I*κ*B-*α* protein and decreased AMP-activated protein kinase (AMPK) phosphorylation in a dose-dependent and time-dependent manner (Ji et al., 2012). Zhou et al. (2014) proposed that genistein inhibited the activation of NF-*κ*B signaling pathway in *β*-amyloid peptide 25–35 (A*β*25-35)-stimulated murine microglial cell line BV-2. The cells were pre-treated with genistein at concentrations of 50 μM for 2 h and then stimulated with 25 μMAβ25-35 for 24 h. Genistein (50 μM) remarkably suppressed mRNA and protein expression of NF-κB p65, NF-κB p50 and toll-like receptor 4 (TLR4) in BV-2 microglia (*p* < 0.05) when compared to Aβ25-35-only group. However, there was no statistical significance found in mRNA expression of NF-κB p50 with treatment of genistein (*p* > 0.05) when compared with other groups (Zhou et al., 2014).

Apart from that, Jeong et al. (2014) highlighted that pretreatment with genistein (50 µM) effectively inhibited LPS-induced NF-κB p65 nuclear translocation in BV2 microglia. Also, genistein (25 and 50 μM) remarkably inhibited LPS-induced increase in the nuclear NF-κB p65 levels and completely restored the degradation of cytosolic IκB-*α* protein (Jeong et al., 2014). In addition, Li et al. (2014) conducted an *in vitro* assay on MH7A cells to investigate the detailed molecular mechanisms of genistein in anti-inflammatory activity. Result revealed that pretreatment of genistein (20 μM) for 2 h inhibited NF-κB signaling pathway in the tumor necrosis factor TNF-*α*-induced MH7A cells. Genistein reduced the phosphorylation of NF-κB p65, IκB*α* and IKK which antagonize IKK/IκB/NF-κB inflammatory pathway. Besides that, genistein inhibited the activation and translocation of TNF-*α*-induced NF-κB from cytoplasm into nucleus in MH7A cells. Genistein treatment also suppressed ROS/Akt/NF-κB pathway and promoted AMPK activation (Li et al., 2014). Also, according to Han et al. (2015), pretreatment of genistein (10, 50 and 100 μM) exhibited prominent inhibitory effect on the expression of NF-κB p65 protein induced by homocysteine (HCY) in a dose-dependent manner. Result highlighted that 100 μM of genistein almost completely abolished the nuclear translocation of NF-κB in ECV-304 cells as compared with HCY-only group (*p* < 0.01), reaching level comparable to the control group (*p* > 0.05) (Han et al., 2015).

According to Smolinska et al. (2018), pretreatment of genistein for 2 h inhibited TNF-α-induced NF-κB nuclear translocation of p65 in human epidermal keratinocyte cell (HaCaT cell) at concentration of 100 µM (Smolinska et al., 2018). In the study of Wang et al. (2019), genistein exerted an inhibitory effect on the phosphorylation of IκB-α and increased expression of NF-κB p65. Genistein at concentration of 100 μM significantly attenuated TNF-α-induced increase in NF-κB level (*p* < 0.01) in HaCaT cells as compared to TNF-α-only group. Furthermore, genistein concentration-dependently suppressed TNF-α-induced phosphorylation of IκB-α at concentrations of 50 and 100 μM (*p* < 0.01 and *p* < 0.001 respectively) as compared to TNF-α-only group (Wang et al., 2019). In a recent study, Zhu et al. (2020) highlighted that genistein at 10 μM effectively abolished the elevation in both expressions of pre-B-cell colony enhancing factor (PBEF) (*p* < 0.001) and nuclear p65 (*p* < 0.001) in LPS-treated MLE-12 cells, while restored the decrease in expression of cytoplasm p65 (*p* < 0.001) as compared to LPS-only group. Interestingly, its activity was comparable with that of the positive control, FK866 (PBEF inhibitor) at concentration of 10 nm (Zhu et al., 2020).

On the other hand, Du et al. (2018) employed an *in vitro* assay to investigate the anti-inflammatory activity of genistein. Western blot analysis was used to detect and analyze the phosphorylation of c-Jun N-terminal kinase (JNK), p38, extracellular signal-regulated kinase (ERK) and IκB. The result indicated that 10 μM of genistein remarkably inhibited LPS-induced activation of mitogen-activated protein kinase (MAPK) and NF-κB with attendant suppression in the phosphorylation of JNK (*p* < 0.05), p38 (*p* < 0.001), ERK (*p* < 0.001) and IκB (*p* < 0.05) in BV2 microglial cells as compared to LPS-only group (Du et al., 2018). A similar outcome was shown by Ganai et al. (2015) through *in vivo* study. In view of that, significant suppression of _d_-Galactosamine (_D_-GalN) induced NF-κB, MAPK (p-38, ERK 1/2) and IKK*α*/*β* expression in male Wistar rats was seen with pretreatment of 5 mg/kg genistein (*p* < 0.05) as compared with _D_-GalN-induced group with the absence of genistein (Ganai et al., 2015). Another *in vivo* study was conducted by Ji et al. (2011) on nonalcoholic steatohepatitis (NASH) rats induced by high fat diet (HFD). Significant inhibition rate (*p* < 0.05) in HFD-induced phosphorylation of JNK and IκB-α expression was achieved with pretreatment of genistein at dose of 4 and 8 mg/kg, while no significant difference (*p* > 0.05) was found in p38 and ERK 1/2 as compared to HFD-only group. Also, genistein significantly inhibited HFD-induced nuclear NF-κB p65 (*p* < 0.05) and restored the degradation of cytoplasmic IκB-α protein (*p* < 0.05 or *p* < 0.01) as compared to HFD-only group (Ji et al., 2011).

In a study conducted by Dijsselbloem et al. (2007), dendritic cells (DCs) were pretreated with 200 μM of genistein for 1 h, followed by stimulation with LPS (1 μg/ml). The result revealed that genistein significantly suppressed NF-κB DNA binding (p65-p50 heterodimers and p50 homodimers) and p65 nuclear localization which induced by LPS in human monocyte-derived dendritic cells (MoDCs), while up-regulated protein levels of p53. Also, the experiment was conducted using HEK293T cell line, which stably expresses TLR4/MD2 proteins. Result demonstrated that genistein (50, 100 and 200 μM) significantly and dose-dependently suppressed LPS-induced NF-κB-dependent promoter activity (*p* < 0.01) in HEK293T cells as compared to untreated cells. To further investigate the effect of genistein on NF-κB, *in vivo* study was conducted using bone marrow dendritic cells (BMDCs) from p53^+/+^ and p53^−/−^ mice. Result revealed that 200 μM of genistein remarkably decreased the p65 nuclear abundance that induced by LPS in p53^+/+^ BMDCs, but no effect in p53^−/−^ BMDCs (Dijsselbloem et al., 2007).

Kim et al. (2011) investigated the anti-inflammatory potential of genistein in angiotensin II-induced NF-κB activation using both *in vivo* and *in vitro* studies. Result revealed that oral administration of genistein (2 and 4 mg/kg) effectively and dose-dependently inhibited (*p* < 0.05) age-related phosphorylation of cytosolic IKK*α*/*β* and IκB-*α*, and restored the degradation of cytoplasmic IκB-α protein (*p* < 0.05) as compared to old, untreated group. Also, genistein significantly and dose-dependently inhibited (*p* < 0.05 or *p* < 0.01) NF-κB nuclear translocation of p65 and p50, and phosphorylation of nuclear p65 subunit (Ser 536) in aged rats as compared to old, untreated group. For the *in vitro* study, similar outcomes were noted in angiotensin II-induced YPEN-1 cells with 1 and 5 µM of genistein (*p* < 0.01) (Kim et al., 2011).

From the *in vivo* study of Lu et al. (2020), the result indicated that genistein inhibited the expression of NF-κB p65, which is induced by chronic sleep deprivation (CSD) in mice at concentrations of 10, 20 and 40 mg/kg. In the cortex of CSD-treated mice, only 10 mg/kg of genistein significantly reduced the protein expression of NF-κB p65 (*p* < 0.05) as compared with CSD-treated only group, which was comparable with that of the positive control, modafinil (MOD) 100 mg/kg. Besides, in the hippocampus of CSD-treated mice, genistein (10, 20 and 40 mg/kg) showed significant inhibitory effects against NF-κB protein expression (*p* < 0.05 or *p* < 0.01) as compared with CSD-treated only group, which was comparable with that of the positive control, modafinil (MOD) 100 mg/kg (Lu et al., 2020).

#### 3.1.2 Prostaglandins Inhibition

The prostaglandins (PGs) are formed when arachidonic acid (AA) is released from tissue phospholipids by the enzyme phospholipases (PLAs) and metabolized by the sequential actions of prostaglandin G/H synthase, or cyclooxygenase (COX), and respective synthases (Sala and Folco, 1991; Ricciotti and FitzGerald, 2011). PG is involved in the generation of the inflammatory response which their biosynthesis is significantly increased in inflamed tissue. (Ricciotti and FitzGerald, 2011). PGE_2_, which synthesized from PGH_2_ by cPGES or mPGES-1 and mPGES-2, is ubiquitously throughout the body and exhibits versatile biological functions (Park et al., 2006; Attiq et al., 2018). It plays an important role in inflammation as it is involved in all processes leading to the classic signs of inflammation, including redness, swelling, and pain (Funk, 2001; Ricciotti and FitzGerald, 2011). Therefore, PG, which contributes to the generation of the inflammatory response, may be the candidate drug target for anti-inflammatory therapy.

According to Akarasereenont et al. (1994), inflammation was associated with the induction of COX-2 activity and protein expression, likely involving the release of main COX metabolites such as 6-oxo-PGF_1α_ and PGF_2α_. It was also revealed that genistein treatment efficiently and dose-dependently downregulated the LPS-induced levels of 6-oxo-PGF_1α_ (0.05–50 μM, *p* < 0.05) in bovine aortic endothelial cells (BAEC) and PGF_2α_ (5–50 μM, *p* < 0.05) in J774.2 macrophages when compared to LPS-only group. Additionally, the results demonstrated that treatment of genistein 50 µM completely inhibited LPS-induced expression of COX-2 protein in both BAEC and J774.2 macrophages (Akarasereenont et al., 1994). Apart from this, there was another study carried out to evaluate the effect of genistein in LPS-treated cultured human chondrocytes. The result showed that treatment of genistein (100 µM) effectively inhibited LPS-induced upregulation of COX-2 protein level (*p* < 0.05) as compared with LPS-only group, but no effect on COX-1 protein level (Hooshmand et al., 2007).

Likewise, Swami et al. (2009) focused on the effect of genistein in prostaglandins pathway through inhibition of COX-2 expression, decrease in PG receptor and suppression in PGE_2_ secretion. Result revealed that genistein (10 μM) significantly suppressed mRNA expression level of COX-2 (PG synthesizing enzyme) in prostate cancer (PCa) cell lines and primary prostatic epithelial cells. At the same dose, mRNA expression of PG receptors (*EP4 and FP*) were significantly reduced in LNCaP cells (*p* < 0.001 and *p* < 0.05 respectively), but no significant effect in PC-3 cells. Also, genistein effectively reduced *EP4* mRNA levels in primary prostatic epithelial cells (E-PZ-1, -2 and -3, and E-CA-1 and -3) (*p* < 0.05–*p* < 0.001). Concomitantly, genistein (10 μM) significantly decreased PGE_2_ secretion in LNCaP cells, PC-3 cells and primary epithelial cell cultures (Swami et al., 2009).

Hämäläinen et al. (2011) carried out an *in vitro* study to investigate the anti-inflammatory activity of flavonoids on PGE_2_ formation, COX-2 and mPGES-1 expression in activated macrophages. The study showed that the twelve flavonoids significantly inhibited PGE_2_ formation, while four of them effectively suppressed COX-2 expression and only two flavonoids markedly inhibited mPGES-1 expression (*p* < 0.01) in LPS-induced J774 macrophages. Among the flavonoids, treatment of genistein (100 µM) showed significant inhibitory effect (*p* < 0.01) against LPS-induced PGE_2_ production (89.8 ± 0.8%), COX-2 mRNA (54.9 ± 5.8%) and protein expression (40.8 ± 7.0%) (Hämäläinen et al., 2011). In the study of Jeong et al. (2014), 25 and 50 μM of genistein significantly and concentration-dependently inhibited LPS-induced PGE_2_ production (*p* < 0.05) in BV-2 microglial cells as compared to LPS-only group. Further study discovered that the genistein also intensively suppressed gene expression in LPS-stimulated BV-2 microglial cells by reduction in COX-2 mRNA and protein level (Jeong et al., 2014). A similar outcome was displayed by dose-dependent inhibition of COX-2 mRNA expression in LPS-stimulated BV2 cells (Du et al., 2018).

In the *in vivo* study by Sutrisno et al. (2017), oral administration of genistein inhibited COX-2 pathway in mice model of endometriosis. Leuprolide acetate and dienogest were set as the positive control in this study. Genistein at dose of 0.78 and 1.3 mg/day significantly decreased the expressions of COX-2 in mice model of endometriosis (*p* < 0.05). Also, genistein (0.78, 1.04 and 1.3 mg/day) portrayed significant inhibitory effect on the expression of PGE (*p* < 0.05) in comparison to the endometriosis group, which was equivalent to that of dienogest. Surprisingly, genistein has a better suppressive effect than the positive control, leuprolide acetate (0.00975 mg) and dienogest (0.0052 mg). Both positive controls decreased the expression of COX-2, but no significant difference as compared with endometriosis group (*p* > 0.05) (Sutrisno et al., 2017).

#### 3.1.3 Pro-Inflammatory Cytokines Inhibition

Proinflammatory cytokines are mainly synthesized by activated macrophages and contribute to the up regulation of inflammatory response. Various pro-inflammatory cytokines including IL-1β, IL-6, and TNF-α are responsible for the process of pathological pain by directly triggering nociceptive sensory neurons (Zhang and An, 2007). There are several studies demonstrate that genistein has suppressive effect on the production of proinflammatory cytokines. These findings could be useful to recognize potential treatment options for chronic inflammatory disorders. In an effort to explore the effect of sophoricoside and its analogs on proinflammatory cytokines, Yun et al. (2000) evaluated the sophoricoside and its analogs (genistin, genistein and orobol) isolated from *Sophora japonica* L. Result suggested that genistein showed significant inhibitory effects against IL-5, IL-3, IL-6 and granulocyte-macrophage colony-stimulating factor (GM-CSF) in a concentration-dependent manner with inhibitory concentration, IC_50_ values of 19.4, 28.4, 13.3 and 59.8 μM respectively. However, it showed no inhibitory effects on both IL-1β and TNF-α (Yun et al., 2000).

According to Ji et al. (2012), genistein (0.1, 1, 5, or 10 μM) effectively and dose-dependently inhibited LPS-induced TNF-α (1–10 μM, *p* < 0.05) and IL-6 (5–10 μM, *p* < 0.05) mRNA levels in macrophages as compared to LPS-only group. Interestingly, the suppressive effect exhibited by 10 μM of genistein was similar to that with the positive control, AMPK agonist 5-aminoimidazole-4-carboxamide-1-*β*-d-ribofuranoside (AICAR) (1 mM) (Ji et al., 2012). On the other hand, Jeong et al. (2014) reported that genistein inhibited TNF-α and IL-1β production via inhibition of gene expression in LPS-stimulated BV-2 cells. Pretreatment of genistein at concentration of 25 and 50 µM effectively suppressed the production of TNF-α and IL-1β which was stimulated by LPS in a concentration-dependent manner (*p* < 0.05) as compared to LPS-only group. Besides that, genistein (25 and 50 µM) reduced the mRNA and protein levels of TNF-α and IL-1β in LPS-induced BV2 microglia. This may indicate that the inhibition of TNF-α and IL-1β production by genistein might be resulted from the inhibition of gene expressions (Jeong et al., 2014). Furthermore, Kim et al. (2014) reported that pretreatment of genistein (12.5, 25 and 50 µM) effectively and concentration-dependently attenuated the phorbol 12-myristate 13-acetate (PMA)/A23187-induced IL-1β and IL-6 gene expression, and IL-6 production in HMC-1, but no effect in TNF-α (Kim et al., 2014).

In the research of Sutrisno et al. (2014), they proposed that genistein exhibited inhibitory effects on proinflammatory cytokines such as IL-1β, TNF-α and IL-6 through *in vitro* model. Genistein at dose of five until 50 μM significantly reduced the level of TNF-α and IL-6 in supernatant cells as compared with control group in all duration of treatment (6, 24 and 48 h) (*p* < 0.05). Besides, significant downregulation (*p* < 0.05) of the level of IL-1β was shown in the culture of endometriosis cells with genistein as compared with control group for 6, 24 and 48 h incubation period (20–50 μM, 5–50 μM and 10–50 μM respectively) (Sutrisno et al., 2014). Meanwhile, Zhou et al. (2014) proposed that genistein at concentration of 50 μM effectively abolished (*p* < 0.05) the elevation in both mRNA and protein expression of proinflammatory cytokines, such as IL-1β and iNOS in Aβ25-35-treated BV-2 cells. Also, genistein significantly restored (*p* < 0.05) the decrease in expression of anti-inflammatory mediator IL-10 at concentration of 50 μM as compared to Aβ25-35-treated control (Zhou et al., 2014). In addition, Li et al. (2014) suggested that genistein (5, 10, 20 µM) exerted concentration-dependent inhibition (*p* < 0.05 or *p* < 0.01) on TNF-α-induced proinflammatory cytokine production such as IL-1β, IL-6, and IL-8 in MH7A cells as compared to TNF-α-only group. Results showed that genistein at concentration of 20 µM possessed stronger inhibitory effect than N-acetyl-l-cysteine (NAC), phosphoinositide-3 kinase (PI3K) inhibitor LY294002 and AICAR in TNF-α-stimulated MH7A cells (Li et al., 2014).

In a study by Choi et al. (2016), the association between the anti-inflammatory effect of genistein and the production of proinflammatory mediators was evaluated in LPS-induced murine macrophages. Pretreament of genistein for 30 min significantly and dose-dependently suppressed the production of proinflammatory mediator IL-6, which was increased by LPS treatment (10 μg/ml). Results highlighted that the secretion of IL-6 was markedly attenuated (*p* < 0.01) by 91% at a dose of 50 µM. In addition, the LPS-stimulated mRNA expression level of IL-6 was also decreased by genistein treatment (5–50 μM, *p* < 0.01) as compared to LPS-treated control, suggesting that genistein inhibited the production of IL-6 through the downregulation of its gene expression (Choi et al., 2016). These findings were supported by the latest work of Du et al. (2018), as the mRNA expression of TNF-α, IL-1β and IL-6 were significantly inhibited (*p* < 0.05–*p* < 0.001) by genistein (10 and 20 μM) in LPS-stimulated BV2 cells as compared to LPS-only group (Du et al., 2018). In the study of Smolinska et al. (2018), 100 µM genistein significantly inhibited levels of IL-8, IL-20, and CCL2 which induced by proinflammatory “cytokine mix” (ACT), TNF-α and LPS in HaCaT cells (*p* ≤ 0.05 or *p* ≤ 0.001), except for IL-20 induced by TNF-α and LPS. Interestingly, most of the inhibitory effect of genistein is stronger than the positive control, methotrexate (1 µM) except for IL-8 induced by TNF-α (3 times potent than genistein) (Smolinska et al., 2018).

Research study of Mace et al. (2019) showed that genistein at concentration of 10 and 25 µM significantly suppressed IFN-*γ* production induced by IL-12/IL-18 in cell culture supernatants from peripheral blood mononuclear cells (PBMCs) (*p* = 0.0023). Besides, 25 µM of genistein significantly reduced IFN-*γ* intracellular staining in CD3^−^NK^Dim^ (open) and CD3^−^CD56^Bright^ (shaded) NK cells (*p* = 0.0153 and *p* = 0.0147 respectively). Also, genistein (25 µM) decreased IL-12/18-induced IL-18Rα expression on CD56 ^+^ NK cells (*p* < 0.01), but no impact on the expression of IL-12Rβ1 (Mace et al., 2019). Widowati et al. (2019) proposed that genistein isolated from *Glycine max* L. Merr exhibited inhibitory effect on the production of TNF-α and IL-1β at concentration of 40 μg/ml in LPS-treated RAW 264.7 cells (Widowati et al., 2019) whereas Zhu et al. (2020) demonstrated that genistein remarkably inhibited LPS-induced increase in TNF-α, IL-1β, IL-6, and keratinocyte-derived chemokine (KC) in a concentration-dependent manner (1 and 10 μM) in LPS-stimulated MLE-12 cells (*p* < 0.001) (Zhu et al., 2020).

In light of the investigation by Dijsselbloem et al. (2007), the anti-inflammatory and immunomodulatory properties of genistein were evaluated in both MoDCs and mice. For the *in vitro* assay, DCs were treated with 200 μM of genistein for 1 h, followed by stimulation with toll-like receptors (TLR) agonists such as LPS (1 μg/ml), polyinosinic-polycytidylic acid [poly (IC)] (10 μg/ml), or FSL1 (100 ng/ml) for 6 h. The result revealed that pretreatment of genistein profoundly represses TLR-dependent IL-6 production as compared to TLR-only groups. Interestingly, LPS promoted the highest secretion of IL-6 among TLR agonists. Hence, the effect of genistein on LPS-stimulated MoDCs was further investigated. Result highlighted that pretreatment of genistein (6.25–200 μM) significantly and dose-dependently inhibited LPS-induced up-regulation of IL-6 in MoDCs with IC_50_ value of 52.07 μM. Also, genistein (200 μM) significantly suppressed LPS-induced IL-6 transcription (*p* < 0.01) in MoDCs without profoundly affecting proximal TLR4-initiated kinase signaling pathways such as LPS-induced IKK, MAPK (ERK, p38, JNK), and mitogen- and stress-activated protein kinase 1 (MSK1) activation. In another account, the effect of genistein on IL-6 expression was also examined through *in vivo* study. Pretreatment of genistein (200 μM) remarkably suppressed LPS-stimulated IL-6 mRNA levels (70% reduction) in the majority of p53^+/+^ BMDCs, but no impact in p53^−/−^ BMDCs (Dijsselbloem et al., 2007).

Also, genistein was evaluated by Babu et al. (2012) to explore the inhibitory effect on hyperglycemia-induced vascular inflammation by using both *in vitro* and *in vivo* systems. For *in vitro* study, the experiment was conducted using the human aortic EC which were cultured with high glucose (25 mmol/L) for 48 h. High glucose was demonstrated to promote the secretion of IL-8 and MCP-1 compared to normal glucose-incubated cells (5.5 mmol/L). However, pretreatment with genistein (1 and 10 μmol/L) for 30 min significantly decreased (*p* < 0.05) the levels of IL-8 and MCP-1. In another account, the effects of genistein on vascular inflammation were examined using diabetic mice. Results demonstrated that dietary intake of 0.1% genistein for 8 weeks effectively reduced the serum concentration of MCP-1, KC, ICAM-1 and VCAM-1 (*p* < 0.05), which were higher in diabetic mice than those in normal mice. By contrast, genistein treatment completely reversed the concentration of IL-10 which was lower in diabetic mice (*p* < 0.05) (Babu et al., 2012). In the recent work, Wang et al. (2019) developed particular interest on the therapeutic potential of genistein on psoriasis-related inflammation. *In vivo* model, it was proposed that topical application of genistein (0.5 and 2%) onto the imiquimod (IMQ)-induced psoriatic mice skin can significantly attenuate the level and mRNA expression of IL-1β, IL-6, TNF-α, IL-17 and IL-23 (*p* < 0.05–*p* < 0.001). Surprisingly, genistein was proved to have a superior inhibition effect than the positive control, Daivonex (calcipotriol ointment). In *vitro* assay, the level and mRNA expression of IL-1β, IL-6, IL-8, IL-23, TNF-α and VEGFA (*p* < 0.05–*p* < 0.001) in TNF-α-treated HaCaT cells were also shown to suppress significantly by 100 μM of genistein except for IL-1β level (*p* > 0.05) (Wang et al., 2019).

In another account, De Paula et al. (2008) examined the clinical and biological effects of genistein on the experimental autoimmune encephalomyelitis (EAE) models. For this *in vivo* study, EAE model was achieved by triggering the clinical disease in C57Bl/6 mice through injection of myelin oligodendrocyte glycoprotein 35–55 peptide (MOG_35–55_). Genistein 200 mg/kg were administered subcutaneously at 14 days post-immunization (dpi) for 7 days. The result demonstrated that genistein significantly suppressed the upregulation of IFN-*γ* (*p* < 0.01), IL-12 (*p* < 0.01), and TNF-α (*p* < 0.001) cytokines, and upregulated the decrease of IL-10 level (*p* < 0.001) in the brain as compared to untreated mice. In contrast, significant inhibition of IFN-*γ* (*p* < 0.05) and IL-10 (*p* < 0.01) cytokines was shown on the splenocyte supernatants of the mice treated with genistein in comparison to untreated mice. Also, in the genistein-treated mice, there was a reduction in TNF-α production on the splenocyte supernatants as compared with untreated group but with no statistical significance (*p* > 0.05). All in all, the result suggested that genistein modulated the inflammatory cytokines and ameliorate EAC clinical signs in mice, suggesting that genistein may serve an important role in inflammatory diseases (De Paula et al., 2008).

In light of the investigation by Ji et al. (2011), it was proposed that genistein markedly suppressed liver inflammation (*p* < 0.05) during nonalcoholic steatohepatitis development with decrease in inflammation score (4 mg/kg, −1.34 and 8 mg/kg, −1.89) as compared to the HFD groups. Concomitantly, intragastrical administration of genistein for 12 weeks significantly and dose-dependently inhibited HFD induced up-regulation of TNF-α and IL-6 levels, and their mRNA expression (4 mg/kg, *p* < 0.05 and 8 mg/kg, *p* < 0.01) in serum and liver of NASH rats (Ji et al., 2011). Besides, Ganai et al. (2015) suggested that pretreatment of genistein (5 mg/kg/day) for 30 days significantly inhibited (*p* < 0.05) _D_-GalN induced up-regulation of TNF-α and IL-1β levels in male Wistar rats as compared to _D_-GalN-only group (Ganai et al., 2015).

In the work of Sutrisno et al. (2018), it was proposed that genistein (1.04 and 1.3 mg/day) significantly reduced the expression of TNF-α and IL-6 in mice model of endometriosis (*p* < 0.05), which was comparable with the positive control, leuprolide acetate (0.00975 mg/5 days) (Sutrisno et al., 2018). In addition, Lu et al. (2020) suggested that genistein (10, 20 and 40 mg/kg) significantly suppressed the level of TNF-α (*p* < 0.05 or *p* < 0.01), IL-6 (*p* < 0.001) and IL-1β (*p* < 0.001) in the serum of CSD mice. Also, treatment of positive control, modafinil (MOD) 100 mg/kg markedly inhibited IL-6 and IL-1β (*p* < 0.001) but no significant difference in the level of TNF-α in serum of CSD-treated mice (Lu et al., 2020).

#### 3.1.4 Inducible Nitric Oxide Synthase Inhibition

Nitric oxide (NO) is a signaling molecule produced from l-arginine by inducible nitric oxide synthase (iNOS) that contributes to the pathogenesis of inflammation (Abramson, 2008; Kumar et al., 2013). Overexpression of iNOS is often been observed in many inflammatory diseases such as asthma and colitis (Kröncke et al., 1998). Hence, iNOS can be a distinct target for drug development in anti-inflammatory therapy. According to the study from Akarasereenont et al. (1994), J774.2 macrophages were treated with LPS (1 µM) and the nitrite level increased from <1 µM in untreated cell to 17.6 ± 0.8 µM. However, for cell treated together with genistein at dose 5 µM (*p* < 0.05), 15 µM (*p* < 0.005) and 50 µM (*p* < 0.005), it showed a dose-dependent inhibitory effect on nitrite accumulation as compared with LPS-only group. Apart from that, 50 µM of genistein had a stronger inhibitory effect than 5 µM erbstatin (Akarasereenont et al., 1994).

In another account, Fuu Sheu et al. (2001) reported that soy isoflavones such as genistein, daidzein and glycitein possessed suppression effects on nitric oxide production in LPS-induced RAW 264.7 cells. Results revealed that 20–100 µM of isoflavone markedly and dose-dependently inhibited (*p* < 0.05) nitrite accumulation as compared to LPS-treated control. Interestingly, genistein with IC_50_ of 50 µM had the strongest inhibitory effect (100 μM, 67.7%) among the isoflavones. To further investigate the underlying mechanism of action, the iNOS activity and expression were examined. Result demonstrated a prominent suppressive effect (*p* < 0.05) on iNOS activity with 100 µM of genistein, with inhibition rate of 36.5%, which is higher than both daidzein (26.7%) and glycitein (19.9%). On the other hand, dose-related attenuation of iNOS protein expression in LPS-stimulated macrophages was observed with the treatment of isoflavones. Among the isoflavones examined, genistein at a concentration of 100 µM had the strongest suppressive effect (89%, *p* < 0.05). Besides, genistein also exhibited significant inhibitory effect (66.4%, *p* < 0.05) against LPS-induced iNOS mRNA expression superior to that of daidzein (57.8%) and glycitein (57.2%) at the doses of 100 µM (Sheu et al., 2001). Furthermore, a similar research was conducted by Choi et al. (2016) with 5 to 5 µM of genistein. The result revealed that pretreatment of genistein remarkably and dose-dependently inhibited NO production, iNOS protein and RNA expression in LPS-induced macrophages (Choi et al., 2016).

Gottstein et al. (2003) examined the effects of isoflavones on nitric oxide production and tumour necrosis factor secretion at dose ranging from 5 to 100 μM. The study demonstrated genistein significantly and dose-dependently inhibited IFN-*γ* plus LPS-induced nitric oxide production (5–100 μM, *p* < 0.05) and tumour necrosis factor secretion (50–100 μM, *p* < 0.05) in RAW 264.7 macrophages with IC_50_ of 57.9 and 52.9 μM respectively. Interestingly, genistein has a superior inhibitory effect than daidzein, where higher concentration of daidzein was necessary to significantly inhibited nitric oxide production (50–100 μM, *p* < 0.05) in LPS-induced macrophages (Gottstein et al., 2003). Also, Hooshmand et al. (2007) showed that genistein significantly inhibited LPS-induced NO production in cell culture supernatants at dose of 50 μM as compared with LPS-treated control cells (*p* < 0.05) (Hooshmand et al., 2007). Furthermore, Hämäläinen et al. (2007) conducted an *in vitro* assay using J774 macrophages. Results highlighted that NO production was remarkably inhibited by genistein in a dose-dependent manner, with an IC_50_ value of 30 μM. Interestingly, its inhibitory effect at dose of 100 μM (97.4%) was comparable with that of the positive controls, NOS inhibitor L-NIO (1 mM) and a selective iNOS inhibitor 1400W (1 mM) (>90%). On the other hand, genistein was proved to have prominent suppressive effect (*p* < 0.01) on both protein and mRNA expression of iNOS at a concentration of 100 μM (Hämäläinen et al., 2007). In another account, Jin et al. (2012) assessed the anti-inflammatory and antioxidant activities of *Pueraria lobata* roots and its active components through an *in vitro* system. Result demonstrated that genistein isolated from *P. lobata* roots showed significant inhibitory effects against LPS-induced NO production with IC_50_ value of 8.08 ± 1.17 µM which was comparable to that of the positive control, 2-amino-5,6-di-hydro-6-methyl-4H-1,3-thiazine hydrochloride (AMT) which was an iNOS inhibitor (IC_50_ of 0.004 ± 0.00 µM) (Jin et al., 2012).

In an attempt to investigate the anti-inflammatory effect of phytoestrogen, Jantaratnotai et al. (2013) conducted a study using highly aggressive proliferating immortalized (HAPI) microglial cells. It has been suggested that pretreatment of genistein at concentration of 0.01, 0.1 and 1 μM for 1 h significantly and dose-dependently suppressed LPS-induced NO production (*p* < 0.05) compared to cell treated with LPS alone, which was similar to that of the positive control, estradiol (0.0001–0.1 μM). Additionally, genistein (1 μM) significantly suppressed LPS-induced upregulation of iNOS, IRF-1 and pSTAT1 protein expression, and iNOS mRNA expression (*p* < 0.05) when compared to cells treated with LPS alone which was comparable with that of the positive control, 0.1 μM of estradiol (Jantaratnotai et al., 2013). On the other hand, Du et al. (2018) employed an *in vitro* study to explore the effect of genistein on inflammatory reaction. In this study, BV2 microglia were treated with 1 μg/ml of LPS for 6 h. The result indicated that under stimulation of LPS, the mRNA expression of inflammatory limited enzymes (iNOS) significantly increase compared with the control cell (*p* < 0.001). However, with the pretreatment of 10 and 20 μM genistein, the upregulation of iNOS was effectively and dose-dependently inhibited in LPS-induced BV2 microglial as compared with LPS-only group (*p* < 0.001) (Du et al., 2018).

Sadowska-Krowicka et al. (1998) had carried out both *in vivo* and *in vitro* study to investigate the protective role of genistein in the pathogenesis of chronic intestinal inflammation through the inhibition of NO formation. In the *in vivo* study, the guinea pigs were treated with trinitrobenzenesulfonic acid (TNBS) to induce ileitis. The myeloperoxidase (MPO) activity was assessed as index of neutrophil infiltration. Result revealed that the MPO activity and nitrite formation were significantly inhibited by 0.1 mg/kg of genistein (*p* < 0.05). Also, treatment of genistein (0.1 mg/kg) effectively reduced positive staining for iNOS and nitrotyrosine and improved mucosal morphology in villus tips of ileum from guinea pigs induced by TNBS. For the *in vitro* study, the result revealed that both genistein (10 and 100 kg/ml) and iNOS inhibitor, NIL (5 mM) markedly inhibited LPS-induced nitrite production in RAW264.7 cells when compared with LPS-treated cell (*p* < 0.05) (Sadowska-Krowicka et al., 1998).

In another account, Tie et al. (2013) suggested that subcutaneously administered of genistein caused marked inhibition on diabetes-induced cutaneous superoxide and nitrotyrosine production in mice across the dose range of 0.2–5 mg/kg (*p* < 0.05). In addition, genistein completely restored the decrease in cutaneous nitrite level in diabetic mice as compared with untreated diabetic mice (*p* < 0.05). Further study discovered that genistein at concentrations of 0.2, 1 and 5 mg/kg intensively suppressed cutaneous iNOS activity in diabetic mice (*p* < 0.05 or *p* < 0.01), while genistein 5 mg/kg significantly increased cNOS activity (*p* < 0.05) (Tie et al., 2013). All in all, these findings provided valuable evidence that genistein have potential anti-inflammatory effects regarding the inhibition of nitric oxide at both gene transcription and translation levels.

#### 3.1.5 Reactive Oxygen Species Inhibition and Free Radical Scavenging Activity

Reactive oxygen species (ROS) are partially reduced metabolites of oxygen with potent oxidizing capabilities. At low concentration, ROS act as signaling molecules that regulate cell growth, the adhesion of cells toward other cells, differentiation, senescence, and apoptosis (Thannickal and Fanburg, 2000; Sharma et al., 2012; Mittal et al., 2014). However, at high concentrations, ROS are deleterious to cells because they oxidize protein and lipid cellular constituents and damage the DNA. The harmful effects of ROS may cause potential biological damage and is termed as oxidative stress. It occurs when the overproduction of intracellular reactive oxygen species (ROS) cannot be neutralized by the antioxidant system (Dröge, 2002; Sharma et al., 2012; Mittal et al., 2014; Aguilar et al., 2016). In such scenario, genistein with ROS inhibitory potential may exhibit potent anti-inflammatory activity. For example, Hsieh et al. (2011) reported that genistein (2, 5 and 10 µM) significantly and dose-dependently suppressed DG-induced intracellular ROS levels in PC12 cells (*p* < 0.05) (Hsieh et al., 2011).

In light of the investigation by Han et al. (2015), it was proposed that pretreatment of genistein (10, 50 and 100 µM) effectively and dose-dependently inhibited the generation of ROS in ECV-304 cells induced by HCY (138.04 ± 16.02, 116.95 ± 14.09, 43.47 ± 9.86 µM, respectively) when compared with HCY-only group (170.12 ± 15.90 µM) (Han et al., 2015). Besides, Bhattarai et al. (2017) also reported the similar activity. Result showed that pretreatment of 50 µM genistein effectively suppressed the cellular ROS level (*p* < 0.05) in human gingival fibroblasts (hGFs), which were increased by LPS treatment (5 μg/ml, *p* < 0.01) (Bhattarai et al., 2017). Another study conducted by Smolinska et al. (2018) demonstrated the anti-inflammatory activity of genistein in HaCaT cells. Genistein at dose of 100 µM significantly inhibited the inflammatory response induced by TNF-α and LPS by the suppression of intracellular ROS (*p* ≤ 0.05) (Smolinska et al., 2018). In a recent study, Liu et al. (2019) demonstrated that genistein at concentration of 10 µM significantly inhibited ROS production (*p* < 0.01) in IL-1β-induced OA chondrocytes as compared with IL-1β only group (Liu et al., 2019).

The extent of the oxidative stress could be exacerbated by a reduction in antioxidant systems. The first lines defense antioxidants including superoxide dismutase (SOD), catalase and glutathione peroxidase (GPx), suppress the formation of free radicals or reactive species (Dröge, 2002; Sharma et al., 2012; Aguilar et al., 2016). For instance, Valsecchi et al. (2011) proposed an *in vivo* study on C57BL/6J streptozotocin (STZ) diabetic mice to investigate the effect of genistein in diabetes mice. The diabetic mice were administered subcutaneously with genistein at doses of three or 6 mg/kg for 3 weeks, starting 2 weeks since STZ injection. Result revealed that genistein 3 mg/kg completely reverted the diabetes-induced increase in reactive oxygen species level (*p* < 0.01) in sciatic nerve as compared with vehicle-treated diabetic mice. Also, similar outcomes were shown by 3 and 6 mg/kg of genistein in both brain and liver of diabetic mice (*p* < 0.05) (Valsecchi et al., 2011).

To further assess the effect of genistein on oxidative stress, antioxidant enzymatic activities and glutathione content were analyzed by Valsecchi et al. (2011) with spectrophotometric and spectrofluorimetric analysis respectively. Result revealed that genistein 3 and 6 mg/kg significantly suppressed malondialdehyde (MDA) level and glutathione reductase (GR) activity in brain of diabetic mice (*p* < 0.05) but no significant impacts on SOD, GPx and catalase. In liver of diabetic mice, genistein 3 and 6 mg/kg completely restored the activity of GPx (*p* < 0.05) while 6 mg/kg of genistein attenuated MDA increase (*p* < 0.05). However, no effect on catalase activity was shown in both 3 and 6 mg/kg of genistein. Unlike the effect of genistein on GR activity in brain, genistein 6 mg/kg further increased the hepatic GR activity (*p* < 0.05) but genistein 3 mg/kg did not modify this increase (Valsecchi et al., 2011).

### 3.2 Toxicology

There are plenty of studies carried out to investigate the pharmacological activities of genistein, while the toxicological aspects of genistein have yet to be determined. Okazaki et al. (2002) carried out a 28-days repeated-dose toxicity study of genistein. The result showed that oral administration of 400 and 1,000 mg/kg genistein exerted endocrine-disrupting effects with slight or mild vacuolation and mucinification of the epithelium in vagina (2 out of 10 female rats in each dose). Besides, significant increase in prolactin level (50.40 ± 22.78 ng/ml) was shown in male rats with genistein at dose of 1,000 mg/kg of body weight as compared with control group (25.58 ± 10.34 ng/ml) (Okazaki et al., 2002).

Apart from that, Kim et al. (2009) carried out developmental toxicological investigation on high dose genistein in zebrafish embryos. In the study, the zebrafish embryos were exposed to genistein (1 × 10^−4^ M, 0.5 × 10^−4^ M and 0.25 × 10^−4^ M) for 60 h at 24 h post-fertilization. In the 30th hour after treatment, the embryos showed decreased heart rate in a dose-dependent manner (*p* < 0.05). Also, the number of hatched embryos and body lengths of embryos decreased dose-dependently after genistein treatment (0.5 × 10^−4^ M and 0.25 × 10^−4^ M) as compared to the vehicle-treated group (*p* < 0.05). For the embryo treated with 1 × 10^−4^ M of genistein, the embryos did not hatch. In the vehicle-treated group, all embryos survived at 60 h after treatment. However, for genistein treated group, only around six out of ten embryos survived in the presence of 0.25 × 10^−4^ M of genistein, and none of the embryos survived in 1 × 10^−4^ M and 0.5 × 10^−4^ M of genistein. Meanwhile, physical malformations such as pericardial edema, yolk sac edema, and spinal kyphosis were observed on embryos that survived after 0.25 × 10^−4^ M genistein-treated embryos. Granular degeneration of myocytes in skeletal muscle and loss and apoptosis of neural cells in brain were also observed on embryos that survived after 0.25 × 10^−4^ M genistein treatment through histopathological examination. All in all, this study showed that high dose of genistein has a potent teratogenic effect (Kim et al., 2009).

In another account, soya isoflavone was evaluated on its toxicological profile by Sarasquete et al. (2018). Potential toxicity of genistein and daidzein was assessed through zebrafish embryos test (ZFET) by exposing zebrafish embryos to genistein or daidzein (1.25, 2.5, 5, 10 and 20 mg/L) at 2–3 h post fertilization (hpf) and monitoring until 96 hpf. There were significant decreased in the hatching success of embryo observed in genistein treated group (10 and 20 mg/L) as compared to control group (*p* < 0.05). The mortality rate of genistein-treated embryos significantly increased at 96 hpf where around 70% of mortality rate was observed in 5 mg/L of genistein and all embryos or larvae were dead in 10 and 20 mg/L of genistein. The median lethal concentration, LC_50_ of genistein determined at 96 hpf was 4.41 mg/L. Also, genistein up-regulated the oestrogen (*esrrb*) and death receptors (*fas*), *cyp1a* transcript levels and most thyroid transcript signals (except for thyroid peroxidase/*tpo*) in control zebrafish embryos-larvae during their development (Sarasquete et al., 2018).

Several studies have found that low-dose genistein (5–15 mg/kg/day) treatment of mucopolysaccharidoses (MPS) III patients has no major side effects and varies in neurocognitive outcomes. Mice with MPS IIIB that were given a high dose of genistein (160 mg/kg/day) had a significant reduction in heparan sulphate buildup and neuroinflammation in the brain, as well as an improvement in behavioral phenotype. To present, not much research has been done on MPS patients who have received high doses of genistein. This study was conducted in order to examine the safety of high dose genistein treatment in MPS patients with neurological impairment. The findings showed that high dose oral genistein therapy appeared to be safe in MPS patients based on preliminary findings, but more testing in a larger randomised placebo controlled trial is needed to confirm safety and efficacy (K. H. Kim et al., 2013).

## 4 Conclusion

In recent years, there is a demand of exploring the bioactive compounds of medicinal plants for healthcare and finding leading compounds that are novel, safe and effective for several diseases in drug discovery and development. Natural compounds and products are in high demand, and their importance should not be overlooked. Genistein was studied and found to be exhibit versatile pharmacological activities. In this review, the focus has been directed in the mechanisms of anti-inflammatory activities of genistein which include NF-κB inhibition, PGs inhibition, pro-inflammatory cytokines inhibition, iNOS inhibition, ROS inhibition and free radical scavenging activity. Currently available *in vitro* and *in vivo* studies have provided evidence to support the uses of genistein on anti-inflammatory activity. However, no human clinical trial has been done to evaluate the therapeutic potential of genistein in inflammation, despite showing promising anti-inflammatory activities in the *in vitro* and animal studies. Thus, further investigation and randomized human clinical trials should be conducted to establish a more evidence-based clinical profile as well as to address the therapeutic potential and safety of the compound in the treatment of inflammatory disorders. Also, the toxicological aspects of the genistein need to be further investigated and taken into consideration during the discovery and development of new agents to design new anti-inflammatory drugs with good safety profile.
